# Adsorption performance of packed bed column for nitrate removal using PAN-oxime-nano Fe_2_O_3_

**DOI:** 10.1186/2052-336X-12-90

**Published:** 2014-06-04

**Authors:** Mahsa Jahangiri-rad, Arsalan Jamshidi, Mohammad Rafiee, Ramin Nabizadeh

**Affiliations:** 1 Department of Environmental Health Engineering, Islamic Azad University, Tehran Medical Sciences Branch, Tehran, Iran; 2 Department of Environmental Health Engineering, School of public health, Yasuj University of Medical Sciences, Yasuj, Iran; 3 Department of Environmental Health Engineering, Alborz University of Medical Sciences, Alborz, Iran; 4 Center for Air Pollution Research, Institute of Environmental Research, Tehran University of Medical Sciences, Tehran, Iran

**Keywords:** PAN-oxime-nano Fe_2_O_3_, Niatrate, Sorption, Packed bed column, Breakthrough curve

## Abstract

A continuous fixed bed study was carried out by using PAN-oxime-nano Fe_2_O_3_ as a sorbent for the removal of nitrate from aqueous solution. The effect of factors, such as flow rate (2, 5 and 7 mL/min) and bed depth (5, 10 and 15 cm) were studied. Data confirmed that the breakthrough curves were dependent on flow rate and bed depth. The adsoption capacities observed in diffent conditions of flow rates (2,5 and 7 mL/min) were 11.65, 24.38 and 25.89, respectively. Thomas model was applied to experimental data to predict the breakthrough curves using linear regression and to determine the characteristic parameters of the packed bed column. Bed depth/service time analysis (BDST) model was used to investigate the effect of bed depth on breakthrough curves. The results showed that Thomas model was suitable for the normal description of breakthrough curve at the experimental condition. The data were in good agreement with BDST model with R^2^ > 0.98. Statistical analyses were performed on fluoride removal obtained from different flow rates using SPSS16 software by applying Kruskal- Wallis test. These findings suggested that PAN-oxime-nano Fe_2_O_3_ in the column structure presents a great potential in removal of nitrate from aqueous solutions.

## Introduction

The principal sources of nitrogen are from nitrogeneous compounds produced by plant and animals or the mining of sodium nitrate for use in fertilizers, and the atmosphere. The most oxidized form of nitrogen is nitrate $\left({\text{NO}}_{3}^{\;-}\right)$[1,2]. World wide, the average intake of nitrate is about 75 to 100 mg/d, of which approximately 80 to 90 percent comes from vegetables. people on a vegetarian diet may consume as much as 250 mg/d of nitrate. Accordingly, drinking water accounts for only 5 to 10 percent of nitrates consumed [3]. However, if the nitrate levels in the water are five times the MCL (10 mg/L), water may supply a person about half the daily diet requirements^3^. Nitrate is of primary concern for infants younger than 6 months of age. Infants are very susceptible to methemoglobinemia, a condition known as “blue baby syndrome.” High nitrate levels that are reduced in the stomach and/or the saliva of an infant to nitrite cause blue baby syndrome. Nitrite in the blood combines with hemoglobin to form methemoglobin, which reduces the capability of the blood to transport oxygen throughout the body. This results in the skin of a baby turning blue and can be fatal [4]. The present MCL in the United States is 10 mg/L as nitrate and Canada has established a maximum acceptable concentration (MAC) of 10 mg NO_3_ (N/L). Due to the fact that nitrate is a stable, highly soluble ion, it is difficult to remove by conventional processes [4]. Present technologies for nitrate removal from water supplies include chemical and biological denitrification [5], reverse osmosis [6], electrodialysis [7], ion exchange [8] and adsoprtion [9]. The process of adsorption of the material through of a fluid mixture flowing in to a packed column has gained great interest in recent years. There is a need to carry out the column studies to assess the required contact time for the adsorbate to achieve equilibrium as the results obtained from the batch studies for the contaminants adsorption studies may not be directly applied for field application in the treatment of polluted water [10]. In the present study, PAN-oxime-nano Fe_2_O_3_ were used for nitrate removal. Continuous adsorption experiments were conducted to understand and quantify the effect of influencing parameters such as, initial floe rates and bed heights on breakthrough curve. BDST model, which offers a simple approach and rapid prediction of adsorber performance, is applied for modelling adsorption of nitrate in PAN-oxime-nano Fe_2_O_3_ column.

## Material and methods

### Preparation and characterization of PAN-oxime-nano Fe_2_O_3_

Hydroxilamine hydrochloride (16 g), sodium carbonate (12 g), and 0.4 g of PAN powder were added to a 250 mL bottle to which 100 mL of deionized water was added and shaken. The reaction was carried out at 70°C for 120 min. After reaction, the resultant was filtered and let to dry. Fe_2_O_3_ was coated on PAN functionalized by adding 0.2 g of selected Fe_2_O_3_ and 100 mL deionized water in a sealed bottle. The solution was shaken at 70°C for 120 min. The resultant was filtered and dried in a vacuum oven at 60°C. PAN functionalized-Fe_2_O_3_ was used as an adsorbent. The characteristics of PAN-oxime-nano Fe_2_O_3_ was studied by XRD, FTIR and SEM in our earlier study [11].

### Experimental procedure

**Figure 1:**
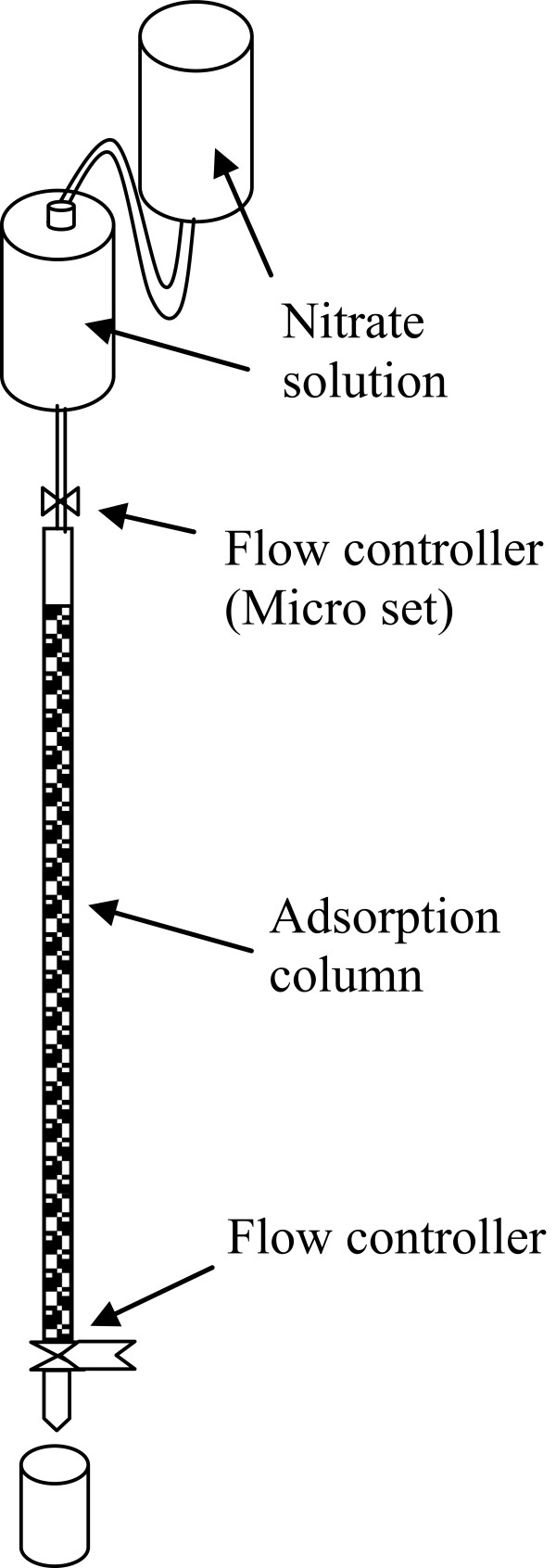
Shematic diagram of packed bed column.

Countinous flow adsorption experiment was conducted in polyethylene columns of 0,5 cm diameter. known quantity of the prepared was packed in the column to yield the desired height of the adsorbent equivalent to 10 g of PAN-oxime-nano Fe_2_O_3_ .At the top of the column the influent nitrate solution (50 mg/l) was entered the packed column (10, 15 and 20 cm) at flow rates of 2,5 and 7 mL/min, using a microset serium. In order to keep the height of nitrate consistent in microset reserviour another feed preservor was installed (Figure 1). The first tank delivers the solution to the second tank at a constant flow rate. The second tank (Micro set) is equipped with a flow controller to help maintain a constant flow rate of the solution being delivered to the column. Smples were collected from the exit of the column at regular time intervals. The samples’ supernatant were separated by centrifugation at 4000 rpm for 10 min. The residual concentration in the supernatants were determined. The nitrate concentration in treated sample were determined by UV–vis Spectrophotometer. The saturation capacity for the PAN-oxime-nano Fe_2_O_3_ was calculated from the following equation:

$$q_{e}=\int_{0}^{V_{e}}\left(C_{0}‒C_{e}\right)\mathrm{dv}/m$$

Where q_e_ is the nitrate adsorbed (mg/g), C_0_ is the influent nitrate concentration (mg/L) C_e_ is effluent nitrate concentration (mg/L), V_e_ is the volume of solution required to reach the exhaustion point (L) and m is the mass of adsorbent (g).

### Modeling of column operation

Full-scale column operation was designed according to the data collected in laboratory level. Many mathematical models have been used for the evaluation of efficiency and applicability of the column models for full scale operations. To design a column sorption process it was necessary to predict the breakthrough curve or concentration time profile and sorption capacity of the sorbent for the selected sorbate. Many models have been developed to predict the sorption breakthrough behaviour with high degree of accuracy. In this study the Thomas model was used to evaluate the behaviour of the selected adsorbent-adsorbate system.

## Results and discussion

### Adsorption capacity of the column

Adsorption capacity of PAN-oxime-nano Fe_2_O_3_ was determined with thomas models. Different volumes of samples containing nitrate (50 mg/L) was passed through the column. The sampling was proceeding until nitrate concentration in outlet reach to nitrate concentration in feed. The adsoption capacities obtained for diffent flow rates(2,5 and 7 mL/min) were 11.65, 24.38 and 25.89, respectively (Tables 1 and 2). The maximum adsorption capacity (q_0_) increased with increase in flow rate.

**Table 1:** Descriptive statistical analysis of various flow rates on fluoride removal at time of 9 h

	**N**	**Mean**	**Std. deviation**	**Minimum**	**Maximum**
Removal	15	85.3333	8.72326	73.00	95.00
Flow	15	4.6667	2.12692	2.00	7.00

**Table 2:** Results of Kruskal Wallis test statistics

	**Removal**
Chi-Square	12.844
df	2
Asymp. Sig.	.002

### Effect of flow rate

**Figure 2:**
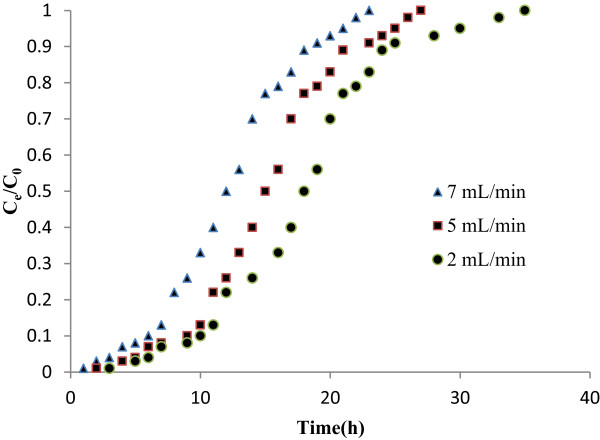
Effect of flow rate on breakthrough curve for nitrate adsorption (bed height 15 cm).

The adsorption columns were operated with different flow rates (2,5 and 7 mL/min) and bed height (10, 15 and 20 cm) untill no further nitrate removal was observed. The breakthrough curve for the column was determined by plotting C_e_/C_0_ against time (Figure 2). The column performed well at lowest flow rate (2 mL/min). As the flow rates and times increased, Earlier breakthrough was observed. The column breakthrough time (C_e_/C_0_ = 0.05) was reduced from 9 to 4 h, as the flow rates increase from 2 to 7 mL/min. This can be due to the two phenomenon: a) a decrease in the residence time which inhibited nitrate contaction to the adsorbent and b) nitrate does not have enough time to diffuse in to the pores of adsorbent and it exited the column without being adsorbed. So at high flow rate the adsorbate solution leavesthe column before equilibrium occures. Similar results was observed in the cases of the copper and nickel rimoval [12]. Thomas model kinetic models was used to analyze the column performance. Thomas model has been used by many researchers to study packed bed adsorption kinetics [13]. The linearized form of the Thomas model is described by equation (1):

(1) $$\text{Ln}\;\left(C_{0}/C_{e}-1\right)=\left(K_{\text{th}}q_{0}M/Q\right)-\left(k_{\text{th}}C_{0}t\right)$$

**Figure 3:**
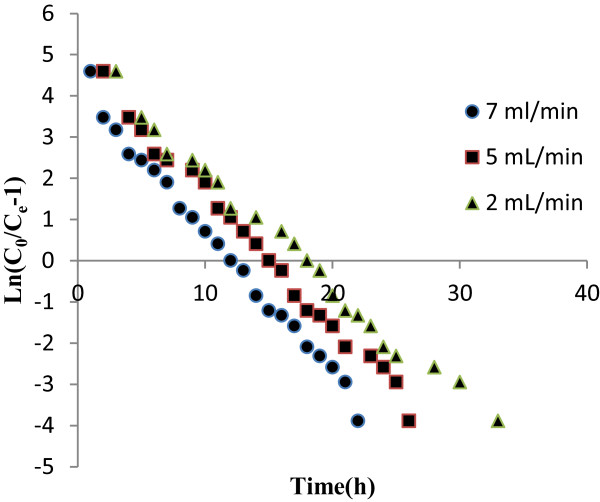
Thomas kinetic plots for the adsorption of adsorption: Effect of flow rate (bed height 15 cm).

Where C_e_, C_0_ = the effluent and inlet solute concentrations (mg/l) at any time (t), qo = the maximum adsorption capacity (mg/g), M = the total mass of the adsorbent (g), Q = volumetric flow rate (mL/h) and K_th_ = the Thomas rate constant (mL/mg h). The kinetic coefficient, Kth and the adsorption capacity of the bed, q_o_ were determined from the plot of Ln [(C_0_/C_e_)-1] against t at a given flow rate (Figure 3). The model parameters are given in Tables 1 and 2. Thomas rate constant, K_th_ is dependent on flow rate. The adsoption capacities obtained for diffent flow rates (2,5 and 7 mL/min) were 11.65, 24.38 and 25.89, respectively (Tables 1 and 2). The maximum adsorption capacity q_0_ increased with increase in flow rate which indicates that the mass transport resistance decreases. The values of K_th_ obtained in this research was similar to the ones obtained by other researchers [14,15]. The Thomas model fitted the experimental data well, with correlation coefficient greater than 0.98, which indicates that the external and internal diffusions are not the rate limiting step. The results obtained from Statistical analysis on fluoride removal in different flow rates is depicted in Tables 1 and 2. By the P value calculated (<0.05) it would be concluded that there is a difference among various flow rate tested. With 2 degrees of freedom, a value of Chi-Square as large as 12.84 is likely to occur by chance only 2 times in a thousand (it has a *p* of 0.002).

### Effect of bed height

**Figure 4:**
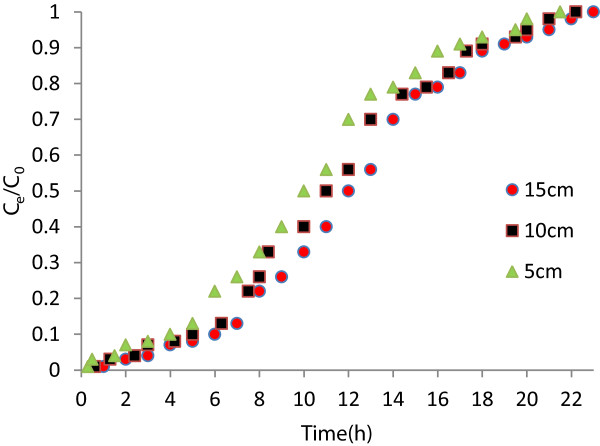
Effect of bed height on breakthrough curve for nitrate adsorption on.

**Figure 5:**
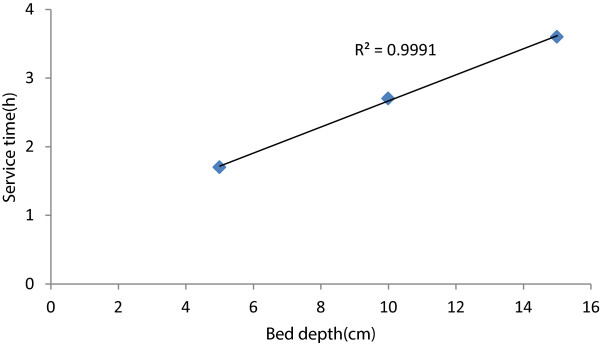
Linear Plot of BDST for nitrate adsorption.

In order to study the effect of bed height on nitrate removal, breakthrough curves for the adsorption of nitrate onto PAN-oxime-nano Fe_2_O_3_ at various bed heights, at the inlet concentration of 50 mg/L and flow rate of 2 mL/min were obtained (Figure 4). The results indicated that the nitrate removal was increased with increase in bed height, due to the availability of more number of sorption sites. At lower bed depth, nitrate ions dont have enough time to adsorbed on adsorbent and a reduction in breakthrough time observed (from 3 to 1 h). It was also observed that the maximum nitrate removal occurs at the initial stage of the experiments. After some time of operation the nitrate removal decreases and reaches to zerowhich might be due to non-availability of sorbent site for the sorption to occur. Another important criteria “breakthrough service time” (BDST) model is used to evaluate the capacity of the bed at various percentage breakthrough values. This model assumes that the adsorption rate is proportional to both the residual capacity of adsorbent and the concentration of the adsorbing solute [16]. This model neglects both the external and internal mass transfer resistances. It is simple, rapid and applicable to predict the effect of different inlet concentrations and bed depth and flow rates on the fixed bed performance. However its validity is limited to a certain range of conditions, for example to a specific range of breakthrough [17]. The BDST model assumes that the service time, ts, for a determined breakthrough concentration, υ, and the height of the bed, Z, are correlated with the process parameters such as maximum adsorption capacity, and rate constant of adsorption in BDST model. The BDST model constants are helpful in determining the full scale process for othe flow rates and adsorbate concentrations without designing new experiments. The BDST equation calculated as follows [18]:

(2) $$t=\frac{N_{o}Z}{C_{o}v}-\frac{1}{K_{a}C_{o}}\ln\left(\frac{C_{o}}{C}-1\right)$$

where C is the breakthrough dye concentration (mg/L), N_0_ is the sorption capacity of bed (mg/L), υ is the the linear velocity (cm/min) and K_a_ is the rate constant (L/mg zmin). The values of BDST parameters are presented in Table. 3. The calculated adsorption capacity (N_0_) and the rate constant (K_a_) are 1433 mg/L and 0.0112 L/mg min, respectively. The value of K_a_ shows the rate of transfer from the fluid phase to the solid phase. When K_a_ is large even a short bed will avoid breakthrough but as K_a_ decreases a deeper bed is required to avoid breakthrough. The advantage of the BDST model is that any experimental test can be reliably scaled up to other flow rates without further experimental runs. The column service time was calculated as the time when normalized concentration was reached. The plot of service time versus bed depth (Figure 5) is linear (R^2^ = 0.999) indicating the validity of BDST model.

**Table 3:** **The Thomas and BDST model parametres for adsorption of nitrate on PAN-oxime-nano Fe**_**2**_**O**_**3**_

**Thomas model parameters**			
**Flow rate (mL/min)**	**q**_**0**_**(mg/g)**	**K**_**th**_**(L/mg h)**	**R**^**2**^
2	11.65	0.0054	0.987
5	24.38	0.0064	0.991
7	25.89	0.0071	0.991
**BDST model parameters**			
**N(mg/L)**	**K**_**a**_**(L/mg h)**	**R**^**2**^	
1433	0.0112	0.956	

## Conclusion

A good removal of nitrate was observed by fixed-bed by PAN-oxime-nano Fe_2_O. The adsorption studied showed that at longer bed depth better removal of nitrate would be achieved. The calculated adsorption capacity (N_0_) and the rate constant (K_a_) were 1433 Mg/L and 0.0112 L/mg h, respectively. Thomas and BDST models were successfully used for predicting breakthrough curves for nitrate removal using different flow rates and depth. The application of the BDST model at 10% of breakthrough point gave satisfactory results with an R^2^ = 0.999.

## Competing interests

The authors declare that they have no competing interests.

## Authors’ contributions

MJ, MR and RN participated in design of the column studies and performed experimental procedures. AJ and MJ participated in statistical analysis. MJ and MR drafted the manuscript. All authors read and approved the final manuscript.
