# Erratum to: Adsorption performance of packed bed column for nitrate removal using PAN-oxime-nano Fe_2_O_3_

**DOI:** 10.1186/s40201-014-0120-8

**Published:** 2014-09-17

**Authors:** Mahsa Jahangiri-Rad, Ramin Nabizadeh

**Affiliations:** Department of environments and energy, Tehran Science and research branch, Islamic Azad University, Tehran, Iran; Center for Air Pollution Research, Institute of Environmental Research, Tehran University of Medical Sciences, Tehran, Iran; Department of environmental health engineering, Tehran university of medical sciences, Tehran, Iran

## Erratum

After publication of this work [1] two authors’ names were removed from the paper. This is due to the fact that the manuscript is a part of a PhD thesis and based on the authorship criteria related to student dissertation the names of the supervisor and student should not be included. This is reflected in the author list of this correction.
